# Curvature Thylakoid1‐like protein CurT mediates thylakoid membrane architecture in *Synechococcus elongatus* PCC 7942

**DOI:** 10.1002/mlf2.70039

**Published:** 2025-10-26

**Authors:** Zimeng Zhang, Xingwu Ge, Tuomas Huokko, Lu‐Ning Liu

**Affiliations:** ^1^ Institute of Systems, Molecular and Integrative Biology University of Liverpool Liverpool UK; ^2^ MOE Key Laboratory of Evolution and Marine Biodiversity, Frontiers Science Center for Deep Ocean Multispheres and Earth System & College of Marine Life Sciences Ocean University of China Qingdao China; ^3^ Present address: Laboratory of Molecular Plant Biology, Department of Life Technologies University of Turku Turku Finland

## Abstract

Photosynthetic electron transfer occurs efficiently in specialized internal membranes known as thylakoid membranes. Thylakoid membranes exhibit diverse structural variations across photoautotrophic organisms. We studied how a key protein, CurT, shapes thylakoid membranes of a model cyanobacterium *Synechococcus elongatus* PCC 7942 (Syn7942), a rod‐shaped cyanobacterium with regular concentric thylakoid layers. By guiding the curves and structure of thylakoid membranes, CurT helps the cells capture light efficiently, especially when conditions change. The detailed characterization of the role of CurT in Syn7942 offers new clues about how nature builds high‐performance photosynthetic membrane systems in response to environmental fluctuations. These findings may inspire future ways to redesign photosynthetic membranes for better crop yields or cleaner bioenergy production.

In chloroplasts and cyanobacteria, thylakoid membranes act as the site of light‐dependent reactions in photosynthesis by housing the photosynthetic machinery, which is composed of the major photosynthetic complexes, including photosystems I (PSI) and II (PSII), cytochrome *b*_6_ *f*, and ATP synthase^1^, ^2^.

Thylakoid membranes exhibit remarkable architectural variations across various photosynthetic organisms^3^, ^4^. In plants and green algae, thylakoids form intricate networks characterized by vertically stacked grana and interconnecting stroma lamellae. This arrangement creates lateral heterogeneity in protein organization, with photosynthetic complexes distributed unevenly between grana and stroma lamellae. Conversely, cyanobacterial thylakoids do not form firmly appressed grana stacks. Instead, they are typically arranged between the plasma membrane and the central cytoplasm, creating highly organized cellular compartmentalization^2^. Cyanobacterial thylakoids display a wide range of configurations, including parietal, radial, coiled, and parallel arrangements^5^.

Despite the architectural variations, thylakoid membranes generally form curved shapes. In chloroplasts, the distinct organization of the grana and stroma lamellae results in a complex architecture with pronounced curvatures, particularly at the grana margins. In the cyanobacterium *Synechocystis* sp. PCC 6803 (Syn6803), thylakoids display a characteristic arrangement of ordered parallel sheets near the cell periphery, complemented by highly curved convergence zones adjacent to the plasma membrane^5^, ^6^, ^7^. The CURVATURE THYLAKOID1 (CURT1) protein family has been recognized as a critical determinant of thylakoid membrane architecture^8^. In *Arabidopsis thaliana*, four CURT1 proteins (CURT1A‐D) have been identified^9^, ^10^. These proteins are localized at the grana margins, and mutations of these proteins led to a decrease in thylakoid phosphorylation and altered membrane curvature, directly influencing the overall thylakoid network structure^10^ and photosynthetic performance, particularly when plants are exposed to stress conditions^11^. Intriguingly, CURT1 homologs have also been found in cyanobacteria. In Syn6803, the single CURT1 homolog, CurT, has been shown to be located at thylakoid convergence zones and plays a crucial role in shaping thylakoid membranes and ensuring the efficient assembly of PSII^6^. A recent study has confirmed that CurT is one of the most abundant proteins co‐purified with FtsH4 that is involved in the biogenesis of PSII in Syn6803^12^.

Compared to the spherical‐shaped Syn6803, the rod‐shaped unicellular cyanobacterium *Synechococcus elongatus* PCC 7942 (Syn7942) forms more regular concentric thylakoid layers^13^, ^14^. The thylakoid membranes are primarily organized as sheet‐like structures arranged in layers parallel to the plasma membrane at the cell periphery, and no physical connection between the thylakoid and plasma membranes has been observed^15^. In this study, we performed extensive characterization of the structure, in vivo localization, and function of CurT in Syn7942. Our data revealed that Syn7942 CurT plays an essential role in inducing thylakoid membrane curvatures, modulating the thylakoid membrane protein profile and lateral distribution of photosynthetic complexes, and regulating photosynthetic performance.

The CurT protein of Syn7942 (Synpcc7942_1832) contains 149 amino acid residues, and its protein sequence shares 37.04% identity and 59.88% similarity with its Syn6803 counterpart (Slr0483)^6^. AlphaFold3‐prediction and sequence analysis revealed that the CurT homologs comprise one amphipathic α‐helix near the N‐terminus (H1), two α‐helices (H2 and H3) in the middle region, and a C‐terminal soluble α‐helix (H4) (Figures 1A and S1A).

**Figure 1:**
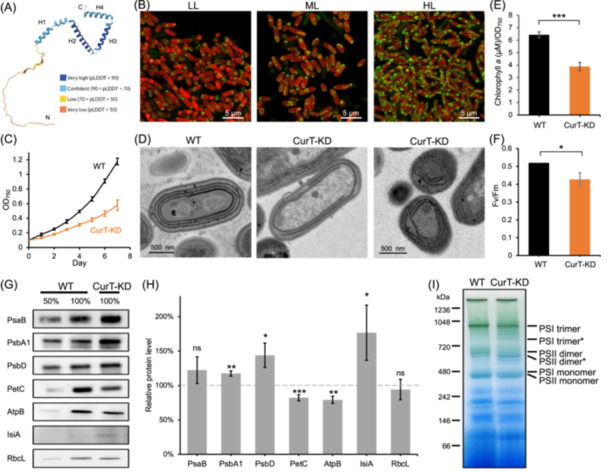
Structural and functional characterization of the CurT protein in Syn7942. (A) A 3‐D model of Syn7942 CurT generated using AlphaFold3. (B) Confocal imaging of the CurT‐GFP strain with fluorescence microscopy under three light conditions: low light (LL), 5 μE·m^−2^·s^−1^; moderate/growth light (ML), 40 μE·m^−2^·s^−1^; high light (HL), 200 μE·m^−2^·s^−1^. (C) Growth curves of the wild‐type (WT) and CurT‐knockdown (KD) Syn7942 strains in BG‐11 medium without antibiotics under constant white illumination (40 μE·m^−2^·s^−1^) at 30°C. (D) Thin‐section transmission electron micrographs of WT and CurT‐KD cells. (E) Chlorophyll *a* content of WT and CurT‐KD using the methanol extraction method, normalized against OD_750_. (F) Fv/Fm measurements (PSII activity) of WT and CurT‐KD (chlorophyll = 20 mM). (G) Immunoblot analysis of extracted thylakoids using annotated antibodies (photosynthetic complexes and RbcL). (H) Quantification of the protein abundance based on western blot results compared to WT (as indicated with the dashed line). Data are shown as means ± SD of three independent experiments. Significant differences were determined with Student's *t*‐test. ns, *p* > 0.05; *, 0.01 < *p* < 0.05; **, 0.001 < *p* < 0.01; ***, *p* < 0.001. (I) Blue native (BN)‐PAGE revealing the oligomeric and monomeric forms of photosynthetic complexes from WT and CurT‐KD. Asterisks designate possible defective complexes.

To examine the structure of Syn7942 CurT, we overexpressed Syn7942 CurT in *E. coli* (Figure S1B). We failed to crystalize CurT for high‐resolution structural determination, probably due to its structurally flexible regions. Circular dichroism (CD) spectroscopy showed negative peaks at 208 nm and 222 nm for ⍺‐helices, a negative peak at 216 nm and a positive peak at 195 nm for β‐sheets, and a negative peak at 200 nm and a positive peak at 212 nm for random coils (Figure S1C). CD data analysis indicated that Syn7942 CurT is predominantly composed of α‐helices and random coils (Figure S1D), consistent with the AlphaFold3‐predicted structure (Figure 1A).

To dissect the in vivo distribution and function of CurT in Syn7942, we fused enhanced green fluorescent protein (GFP) to the C‐terminus of CurT at the native chromosomal locus regulated by the native *curT* promoter, which ensured the expression of CurT in natural context at physiologically relevant levels. We then employed confocal fluorescence imaging on the Syn7942 CurT‐GFP cells grown under different light conditions: low light (LL, 5 μE·m^−2^·s^−1^), moderate light (ML, 40 μE·m^−2^·s^−1^), and high light (HL, 200 μE·m^−2^·s^−1^) (Figure 1B). Under LL, CurT was mainly associated with thylakoid membranes, confirming that it is an integral membrane protein, consistent with the protein model in Figure 1A. Interestingly, it exhibited an uneven distribution along the thylakoid membranes, particularly at the poles and centers of cells where cell division appeared to occur (Figure S2A). This unique subcellular localization suggests that CurT in Syn7942 may play a role in inducing membrane curvature (predominantly at cell poles and cell‐division sites). Additionally, it may also be involved in cell division.

Notably, elevating light illumination during cell growth resulted in a marked increase in CurT‐GFP fluorescence, which was distinctly visible along the thylakoid membranes and occasionally traversing the cytoplasm (Figure 1B). Under HL, CurT proteins were accumulated not only at polar and central locations but also along the lateral regions of the thylakoid membranes (Figures 1B and S2B).

To investigate the exact role of CurT in Syn7942, we sought to delete the *curT* gene from the Syn7942 genome. However, after several attempts, we failed to obtain fully segregated ∆*curT* mutants in any subculture, even at increased concentrations of antibiotics. This suggests that CurT is essential, and that the lack of CurT is lethal in Syn7942^16^, ^17^. Nevertheless, the deletion led to generation of a *curT*‐knockdown mutant (CurT‐KD, Figure S3A). Interestingly, SDS‐PAGE and immunoblot analysis showed that under antibiotic pressure, the abundance of CurT in CurT‐KD fell below the detection threshold compared to the strong immunoblot signal of CurT detected in the wild‐type (WT) strain (Figure S3B). Therefore, we used this knockdown strain to explore the in vivo function of Syn7942 CurT in subsequent studies.

CurT‐KD exhibited a lower growth rate than WT under ML in BG‐11 medium in the absence of antibiotics (Figure 1C). Furthermore, thin‐section transmission electron microscopy (TEM) revealed that the WT Syn7942 exhibited mostly intact thylakoid membranes with concentric and smoothly curved structures, whereas the loss of CurT induced more angular bends and the lack of natural curvature (Figure 1D), indicating its role in determining thylakoid membrane curvature in Syn7942. The loss of membrane curvature has also been observed in Syn6803 cells deficient in CurT^6^. Moreover, typical CurT‐KD cells had fewer thylakoid membrane layers (Figures 1D and S3C) and reduced center‐to‐center distance between neighboring thylakoid membranes (Figure S3D) compared to WT cells. These findings support the role of Syn7942 CurT in maintaining thylakoid membrane curvature and spacing, which are likely correlated with photosynthetic electron transport^18^.

We then conducted physiological measurements of CurT‐KD in comparison with WT. The CurT‐KD cells exhibited a marked reduction in phycobilisomes and chlorophyll *a* content relative to WT (Figures 1E and S4A). Additionally, a blue shift in absorption was observed for the CurT‐KD strain, likely attributed to the presence of IsiA^19^, ^20^ and/or reduced amount of PSI trimers. Fluorescence emission spectra stimulated at 430 nm (chlorophylls) showed that CurT‐KD exhibited higher PSII fluorescence and lower fluorescence at 720 nm than the WT, suggesting a relatively weaker energy transfer to PSI (Figure S4B). The blue shift at 685 nm confirmed the presence of IsiA in CurT‐KD^19^ (Figure S4C). Collectively, CurT deficiency impaired cellular homeostasis, as evidenced by stress‐induced IsiA expression, decreased PSI trimer abundance, and reduced chlorophyll content in Syn7942 cells.

The CurT‐KD strain exhibited a maximum quantum efficiency of PSII (Fv/Fm) of 0.43 ± 0.04, lower than that of WT (0.52 ± 0.01) (Figure 1F), indicating photosynthetic stress triggered by CurT deficiency. PSII quantum yield was notably lower in CurT‐KD relative to WT (Figure S4D), implying increased stress and potentially explaining the elevated CurT levels observed under HL (Figure 1B). A modest increase (although not statistically significant) in the oxygen‐evolution rate and similar respiratory rate were observed in CurT‐KD compared to WT (Figure S4E).

We performed immunoblotting analysis to evaluate whether CurT deficiency alters the composition of thylakoid membrane proteins. The level of PsaB, a PSI core component, was slightly greater in CurT‐KD than in WT (Figure 1G,H). The abundance of PSII subunits PsbA1 and PsbD was increased, probably suggesting a physiological response to affected PSII assembly^6^. In contrast, CurT‐KD exhibited a remarked reduction in cytochrome *b*_6_ *f* (PetC) and ATP synthase (AtpB). IsiA showed an approximately twofold increase, consistent with the blue‐shifted absorption peak from 680 to 673 nm (Figure S4A). The cytosolic large subunit of Rubisco (RbcL), used as an internal reference, exhibited no substantial variation between strains, indicating that CurT deficiency had minimal impact on the Calvin cycle occurring in the cytoplasm.

Blue native‐PAGE (BN‐PAGE) revealed that in CurT‐KD cells, the abundance of fully assembled PSI trimers was reduced, accompanied by an increase in partially assembled trimers, while monomer levels remained comparable to those in the WT (Figure 1I). These results suggest cellular stress, consistent with the shifted absorption peak (Figure S4A). Additionally, CurT‐KD showed smaller PSI trimers and PSII dimers compared to WT (Figure 1I), indicating incomplete PSI and PSII assembly. Overall, CurT knockdown impeded both PSI and PSII oligomer assembly, and the increased levels of individual photosystem subunits (Figure 1G,H) may be a compensatory response to this impairment.

Confocal microscopy and data analysis further indicated that the loss of CurT resulted in a decrease in the quantity of assembled PSI and PSII complexes and a more heterogeneous distribution of both photosystems in thylakoid membranes (Figure S5).

In conclusion, our results showed that Syn7942 CurT proteins were distributed along the thylakoid membranes, and were particularly concentrated at the cell poles and division sites. Moreover, the increase in CurT expression and altered in vivo localization under high light conditions indicates its involvement in light‐dependent thylakoid membrane remodeling (See Supplementary Discussion in Supporting Information). Our findings provide insights into the role of CurT in thylakoid membrane organization and curvature in Syn7942 and highlight the natural modulation of thylakoid architecture for regulating photosynthetic efficiency. This structural‐functional coupling may represent a strategy to balance light harvesting with redox homeostasis under fluctuating environmental conditions. From a broader perspective, our findings highlight the importance of membrane morphology in dictating the photosynthetic organization and efficiency. By harnessing curvature‐inducing elements like CurT, it may be possible to spatially organize electron transport components more effectively, enhancing light capture and reducing excitation pressure. Such strategies could be applied to develop high‐efficiency photobioreactors, biomimetic photosynthetic systems for bioenergy applications by engineering the thylakoid membrane architecture in cyanobacteria or chloroplasts.

## AUTHOR CONTRIBUTIONS

**Zimeng Zhang**: Conceptualization; investigation; methodology; formal analysis; validation; writing—original draft; writing—review and editing. **Xingwu Ge**: Investigation; methodology; visualization; writing—review and editing. **Tuomas Huokko**: Methodology; writing—review and editing. **Lu‐Ning Liu**: Conceptualization; data curation; formal analysis; funding acquisition; investigation; project administration; supervision; validation; writing—original draft; writing—review and editing.

## ETHICS STATEMENT

This study did not involve human subjects and animals.

## CONFLICT OF INTERESTS

The authors declare no conflict of interests.

## Supporting information

Supplementary CurT R3.

## Data Availability

The supporting data are available in Supplementary Information. Any additional raw data will be made available by the corresponding author upon reasonable request.
