# Supplementary material for: Curvature Thylakoid1‐like protein CurT mediates thylakoid membrane architecture in Synechococcus elongatus PCC 7942
Source: mLife. 2025 Oct 26;4(5):567–71. doi: 10.1002/mlf2.70039 (PMC12575082; doi:10.1002/mlf2.70039)
Supplement: Supplementary file 1 — Supplementary CurT R3. [file MLF2-4-567-s001.docx]

**Supplementary Information**

**Materials and Methods**

**Strain growth and construction**

*Synechococcus elongatus* PCC 7942 (Syn7942) was cultured in BG-11 medium at 30°C under continuous white light (40 μE·m^-2^·s^-1^) in Nunc™ Cell Culture Treated TripleFlasks™, with constant agitation. To localize Syn7942 CurT *in vivo*, cells were exposed to three distinct light intensities: low light (5 μE·m^-2^·s^-1^), moderate growth light (40 μE·m^-2^·s^-1^) and high light (200 μE·m^-2^·s^-1^). Fluorescently labeled and knockdown/knockout strains were constructed using the Redirect recombination strategy^1,2^. Briefly, GFP or knockout cassettes were inserted at the 3' end of the target genes, following established protocols^3^. The target gene was amplified by PCR with primers designed to anneal 800 bp upstream and downstream of the gene. The amplified fragment was then cloned into the pGEM-T easy vector, which contains an antibiotic resistance marker. The recombinant plasmid was then used to transform *Synechococcus* cells. For antibiotic selection and maintenance, 50 μg∙mL^-1^ spectinomycin was supplemented in BG-11 for knockout/knockdown strains and 50 μg∙mL^-1^ apramycin was supplemented for GFP strains. Integration of the fluorescent or knockout cassette was confirmed by PCR and sequencing.

**Thylakoid membrane isolation**

Thylakoid membranes were isolated at the exponential growth phase (OD_750_=0.8). A 40 mL culture was centrifuged at 5,000 g for 10 minutes at 4°C, and the resulting pellet was washed in 10 mL of washing buffer [50 mM HEPES (NaOH, pH 7.5), 30 mM CaCl_2_]. The Pellet was then resuspended in 200-300 μL of resuspension buffer [50 mM HEPES (NaOH, pH 7.5), 30 mM CaCl_2_, 800 mM sorbitol, 1 mM 6-aminohexanoic acid] and mixed with an equal volume of glass beads (150-212microns, Sigma G-1145). Cell disruption was performed by vortexing the mixture for 1 minute, repeated six times at 4°C with 1-minute cooling intervals between each round. After vortexing, the sample was centrifuged at 3,000 g, 4°C for 2 minutes, and the supernatant was collected. The remaining pellet was re-extracted by vortexing again with an additional 0.5 mL of resuspension buffer. The combined supernatants were then centrifuged at 18,000 g for 30 minutes at 4°C to pellet the thylakoid membranes. The final thylakoid pellet was resuspended in the resuspension buffer for further analysis.

**Heterologous protein expression and purification**

*Escherichia coli* strains were cultivated in LB medium for protein expression. The wild-type CurT (Syn7942_1832) was PCR-amplified and cloned into the pETDuet expression vector, incorporating an N-terminal Hexa-His tag. The resulting plasmid was introduced into *E. coli* BL21(DE3) cells via heat shock, and the cells were subsequently grown in LB medium containing 100 μg mL^-1^ ampicillin.

For expression, 30 mL of overnight culture was inoculated into 3 L of fresh LB broth and incubated at 37°C with shaking (160 rpm) until the optical density (OD_600_) reached 0.6–0.8. Protein expression was induced by adding IPTG to a final concentration of 0.5 mM, and the culture was kept at 37°C overnight. Cells were harvested by centrifugation and resuspended in phosphate-buffered saline (PBS buffer) (8 g L^-1^ NaCl, 0.2 g L^-1^ KCl, 1.44 g L^-1^ Na_2_HPO_4_, 0.24 g L^-1^ KH_2_PO_4_, pH 7.4). Cell disruption was carried out using a French Press (Stansted Fluid Power, UK) at 110 kPa for 6 cycles. The debris was removed by centrifugation at 3,000 g for 10 minutes at 4°C. The resulting supernatant was then subjected to ultracentrifugation (100,000 g for 30 minutes at 4°C) to separate membrane and cytoplasmic fractions.

Protein purification was conducted using the ÄKTA pure protein purification system (GE Healthcare) and the supernatant was collected and was supplemented with1.5% β-DDM. The sample was subsequently passed through a HisTrap™ High-Performance Column (GE Healthcare) equilibrated with a binding buffer composed of 30 mM imidazole, 0.03% β-DDM in PBS. Bound proteins were eluted with a linear gradient of 0–100% elution buffer containing 500 mM imidazole, 0.03% β-DDM in PBS. Eluted fractions were analyzed by SDS-PAGE, and those enriched in CurT were further purified using Sephacryl high-resolution size-exclusion chromatography resins (GE Healthcare). The purified protein peaks were collected and analyzed for purity. For long-term storage, bacterial strains were mixed with 25% glycerol as a cryoprotectant, snap-frozen in liquid nitrogen, and stored at -80°C.

Proteins separated by SDS-PAGE were transferred onto polyvinylidene difluoride (PVDF) membranes using electrotransfer at 90 V for 45 minutes in transfer buffer (0.29% glycine, 0.58% Tris, 20% methanol). After transfer, the PVDF membranes were rinsed in TBS buffer (20 mM Tris-HCl, 150 mM NaCl, pH 7.5) and blocked with 5% (w/v) milk in TBS for 1 hour at room temperature to prevent nonspecific binding. Following blocking, membranes were washed twice with TTBS buffer (TBS containing 0.1% Tween 20) for 5 minutes with gentle shaking (90 rpm). The membranes were then incubated with primary antibodies (Supplementary Table 1) diluted in TBS buffer containing 1% (w/v) milk powder at room temperature for 3 hours with continuous shaking. After incubation, the membranes were washed four times with TTBS to remove unbound antibodies. Secondary antibody incubation was performed for 1.5 hours at room temperature, followed by sequential washes—three times with TTBS and twice with TBS, each for 5 minutes. Signal detection was carried out using Clarity Western ECL substrate (Bio-Rad), and images were captured with an ImageQuant LAS 4000 imaging system (GE Life Sciences). The α-CurT antibody was kindly provided by Dr Jörg Nickelsen (Ludwig-Maximilians-Universität München)^4^. Antibodies against PsaB, PsbA1, PsbD, PetC, AtpB, IsiA, and RbcL were sourced from Agrisera.

**Blue native-polyacrylamide gel electrophoresis**

Isolated thylakoid membranes were first washed with a washing buffer (330 mM sorbitol, 50 mM BisTris, pH 7.0), and then resuspended in a buffer containing a mixture of equal parts resuspension buffer (25 mM BisTris-HCl, 20% glycerol, 0.025% Pefabloc w/v, 10 mM MgCl_2_, 4% PromegaRNase-Free DNase RQ1) and solubilization buffer (same composition with additional 3% n-dodecyl-beta-maltoside). Membrane suspensions were incubated on ice (4°C) for 30 minutes, followed by centrifugation at 18,000 g, 4°C for 15 min. The resulting supernatant was combined with 10% loading buffer (0.5% SERVA G, 0.5 M 6-amino-caproic acid, 50 mM BisTris-HCl pH7.0, 30% sucrose) and loaded onto a 3-12% linear gradient blue native gels (Invitrogen, Nativepage).

**Chlorophyll concentration**

Chlorophyll quantification was determined using 1 mL of cyanobacterial culture, which was centrifuged at 15,000 g for 7 min at room temperature. The resulting pellet was resuspended in 1 mL of precooled (4°C) methanol and was homogenized. To facilitate pigment extraction, the samples were protected from light by covering them with aluminum foil and incubated at 4°C for 20 minutes. Following incubation, the samples were centrifuged at 15,000 g, for 7 min at 4°C and the supernatant was collected for absorbance measurements at 665 nm and 720 nm. The chlorophyll *a* concentration was calculated using the formula: Chla (µg mL^-1^)= 12.9447 × (A_665_ − A_720_)^5^, Chla (µM) = 14.4892 × (A_665_ − A_720_) for Chla molar mass = 893.4890 g mol^-1^.

**Room temperature absorption and fluorescence spectra**

Whole-cell absorption spectra were recorded at room temperature using a Cary UV-Vis Spectrophotometer (Agilent Technologies). The cell concentration was adjusted to an optical density of OD_750_=1 before measurements. Spectra were collected across the wavelength range of 400 to 800 nm and normalized to the absorbance at OD₇₅₀ to ensure comparability between samples.

Fluorescence emission spectra of whole cells were obtained at room temperature using a Varian Cary Eclipse Spectrofluorometer. The cell density was standardized to OD_750_=1. Excitation wavelengths were set at 430 nm for chlorophyll *a* and 580 nm for phycobilisomes, with an excitation slit width of 5 nm. Emission spectra were recorded from 600 to 780 nm and normalized to the absorbance at OD_750_.

**Measurement of PSII Activity and rapid light curves**

Photosystem II (PSII) activity, represented by the Fv/Fm ratio, and rapid light response curves were assessed using an AquaPen-C fluorometer (Photon Systems Instruments, Brno, Czech Republic) following established protocols^6^. The culture was harvested at OD_750_=0.8 and concentrated to a chlorophyll *a* level of 20 μM. Triplicate 4 mL samples were dark-adapted for 10 minutes before Fv/Fm and light curve measurements were conducted. Data acquisition was facilitated through the AquaPen-C software suite.

**Oxygen-evolution and respiratory rates**

The rates of oxygen evolution and cellular respiration were determined using an OXYLAB2 liquid-phase oxygen electrode (Hansatech). One milliliter of culture was adjusted to a chlorophyll *a* concentration of 20 μM and incubated in a temperature-controlled (30°C) chamber with continuous mixing via a magnetic stirrer. Prior to measurements, samples underwent dark adaptation for 2 minutes. Oxygen evolution was recorded under a 650 nm red LED light, while respiration rates were determined in darkness. Data were captured during steady-state oxygen fluctuation.

**Circular dichroism** **spectroscopy**

Circular dichroism (CD) spectra of purified CurT (7.288 mg mL^-1^) in PBS buffer supplemented with 0.03% n-Dodecyl-β-D-maltosid (β-DDM) using J-1100 Circular Dichroism Spectrophotometer (JASCO) equipped with a PM-539 detector at 20°C. Spectral data were analyzed using the DichroWeb database^7-9^ and processed with CONTINLL algorithms for secondary structure estimation^10,11^.

**Transmission electron microscopy (TEM) analysis**

Syn7942 cells were collected, postfixed with 2% osmium tetroxide, dehydrated through an ethanol gradient (30 to 100%), embedded in resin and ultrathinsectioned (70 nm) as described in previous studies^12-15^. Sections were stained with 1% uranyl acetate and 3% lead citrate before imaging with a Tecnai G2 Spirit BioTWIN transmission electron microscope (FEI) coupled with a Gatan Rio 16 camera. The inter-thylakoid membrane spacing was quantified using FIJI image processing software (ImageJ, NIH).

**Confocal fluorescence microscopy**

Exponentially growing Syn7942 cultures in BG-11 medium were immobilized on a 1.5% BG-11 agar layer and equilibrated for 15 minutes before imaging. A 1 cm × 1 cm agar section was transferred onto a 0.17 mm cover glass. Imaging was conducted at 30°C using a Zeiss LSM710 or LSM780 inverted confocal microscope equipped with a 100x oil-immersion objective and a 2 μm pinhole. GFP fluorescence was visualized with a 488 nm excitation wavelength, with emission detection at 500-520 nm for GFP and 670-720 nm for chlorophyll *a*. Zen 2010 software was used to capture images, ensuring pixel values remained below saturation.

**Image analysis**

Image analysis was conducted using ImageJ software. To quantify protein abundance from immunoblot images^16^, images were inverted and integrated density was measured after background subtraction (rolling ball radius = 50 pixels). Band intensities were compared quantitatively. Thylakoid membrane spacing was analyzed by inverting TEM images, generating a plot profile of grey values, and measuring peak-to-peak distances. Fluorescence distribution and intensity were assessed by selecting thylakoid membranes, generating plot profiles, and analyzing the extracted data. Fluorescence heterogeneity was characterized by calculating the standard deviation of intensity values normalized to the mean.

**Structural analysis and prediction**

Protein secondary structure analysis was performed using the hierarchical protein structure prediction server I-TASSER^17-19^. 3D-structure prediction for CurT was performed by using AlphaFold3^20^. The amino acid sequence of CurT was input into the AlphaFold3 server. AlphaFold3’s deep learning model then predicted the full protein structure by considering the interatomic distances and angles within the sequence. The top-ranked model was selected based on the predicted local and global confidence scores (pLDDT) and the structural alignment against known templates{Jumper, 2021 #409;Jumper, 2021 #409}.

**Supplementary Discussion**

For membrane proteins, individual α-helices or β-sheets are typically markedly longer than those in soluble proteins^21^. Syn7942 CurT contains shorter α-helices than canonical membrane-spanning proteins and has distinct hydrophilic domains, suggesting its classification as an amphipathic protein. Numerous proteins featuring amphipathic helices have been found to play roles in various membrane-related functions, including sensing and generating membrane curvature and facilitating the transport of vesicles within cells. Examples of such proteins include amphiphysin, Arf proteins, epsin, and endophilin^22-25^. The amphipathic helices of these proteins can be inserted into lipid membranes, acting as molecular wedges with varying lipid-binding affinities to contribute to the reshaping of membranes as well as the formation, transport, and fusion of vesicles within cells. In Syn6803, CurT was localized on the convex side of the thylakoid membranes, and deletion of CurT led to the reshaping of thylakoid membranes and loss of canonical thylakoid biogenesis centers, indicating its role in maintaining normal thylakoid membrane architecture^4^.

Despite repeated attempts, a fully segregated Δ*curT* Syn7942 mutant was not generated, in agreement with previous studies indicating that CurT is an essential gene in Syn7942^26,27^. The knockdown of CurT led to alterations in thylakoid membrane morphology, including decreased curvature and fewer layers. These results confirmed that CurT is crucial in Syn7942. Additionally, the absence of CurT disrupted photosystem assembly, with increased levels of PSI and PSII subunits in the CurT-KD strain likely representing a compensatory response. In contrast, CurT appeared to be non-essential in Syn6803 under glucose-supplemented conditions, where its deficiency led to reduced photosystem levels^4^. Notably, the absence of CURT1 proteins did not impact the accumulation of photosynthetic complexes in *A*. *thaliana*^28^. These discrepancies underscore the functional variations in the CURT1 family proteins across different photosynthetic organisms. Moreover, CurT in Syn6803 was specifically located at the thylakoid convergence zones^4^. Conversely, Syn7942 thylakoid membranes are relatively planar and lack apparent thylakoid convergence zones^29^, and CurT proteins were distributed throughout the thylakoid membranes, with a notable concentration at the cell poles and division sites. Analysis of the Syn7942 CurT-KD strain further revealed substantial alterations in the thylakoid membrane structure, including reduced membrane curvature, fewer thylakoid layers, and decreased inter-thylakoidal distances. These structural changes caused by CurT efficiency correlated with modifications in photosynthetic performance, as evidenced by alterations in chlorophyll content and energy transfer efficiency, indicating that proper thylakoid membrane curvature is essential for optimal light harvesting and balanced energy distribution between photosystems. Additionally, previous studies demonstrated that the spacing between thylakoid membranes is subject to physiological regulation^30^. It is plausible that CurT may modulate thylakoid membrane formation and organization, either directly or indirectly, thereby influencing the overall photosynthetic efficiency and adaption.

Interestingly, our recent study showed that Syn7942 CurT was not detected in pull-down assays of photosynthetic complexes^31^, in contrast to previous results in *A*. *thaliana*^32^ and Syn6803^33^, which suggested that CurT is a subunit of PSI. This further implies that Syn7942 CurT is likely not a permanent constituent of photosynthetic complexes, and may not form stable and direct interactions with these complexes.

To date, there has been no evidence of direct fusion between thylakoid membranes and the plasma membrane in cyanobacteria. Recent studies suggested that the anchor protein of convergence membranes (AncM) was located at the “thylapse” regions and interacted with CurT for anchoring the thylakoid membranes towards the plasma membrane at thylapses in Syn6803^34^. However, a homolog of the AncM protein is absent in Syn7942, raising the possibility that CurT functions either as a curvature scaffold on its own or in conjunction with yet unidentified interaction partners.

**
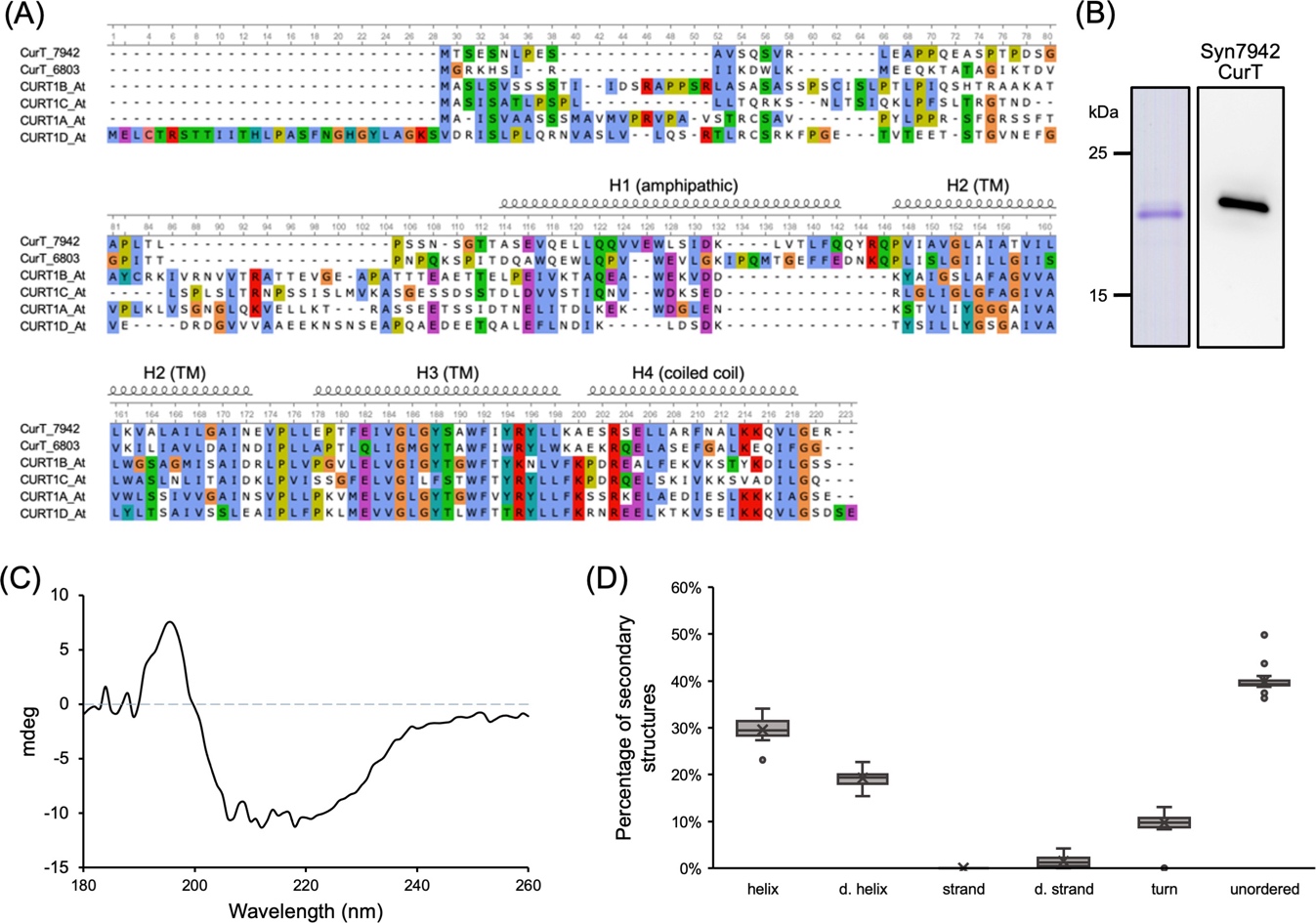
**

**Figure S1. Structural characterisation of the Syn7942 CurT protein. (A)** Comparison of CurT-family protein sequences and prediction of secondary structure using I-TASSER. (**B)** Purification of Syn7942 CurT (with His-tag affinity chromatography and size exclusion chromatography), as determined by SDS-PAGE (left) and immunoblot analysis using an anti-His-tag antibody (right). (**C)** Circular dichroism spectra of purified Syn7942 CurT. (**D)** Percentage of secondary structures analyzed based on circular dichroism data, including α-helix—α- and 3_10_-helices (helix), distorted α-helix (d.helix), β-strand (strand), distorted β-strand (d.strand), turn (turns and bends) and unordered (non-continuous residues assigned to a secondary structure). Data were obtained from the Dichroweb database using COTIN method. Data are shown as means ± SD of the top 35 models.


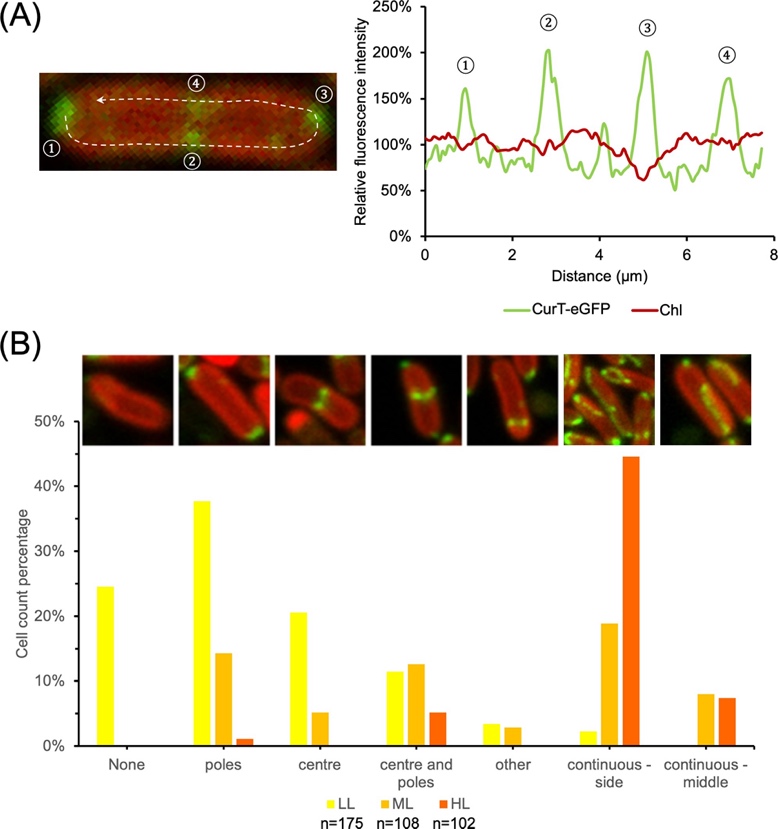


**Figure S2. Location of CurT proteins in Syn7942. (A)** Plot profile of GFP and chlorophyll *a* fluorescence intensity in a CurT-GFP cell, normalised with the mean of fluorescence intensity. Each peak of GFP fluorescence intensity corresponds to a spot with strong GFP signals. (**B)** Relative frequency of individual CurT distribution types under different light conditions. Cells were categorised into groups based on whether the CurT-GFP signals formed dots or lines, and where the signals are located in each cell. Confocal images of typical cells of each group are illustrated above.


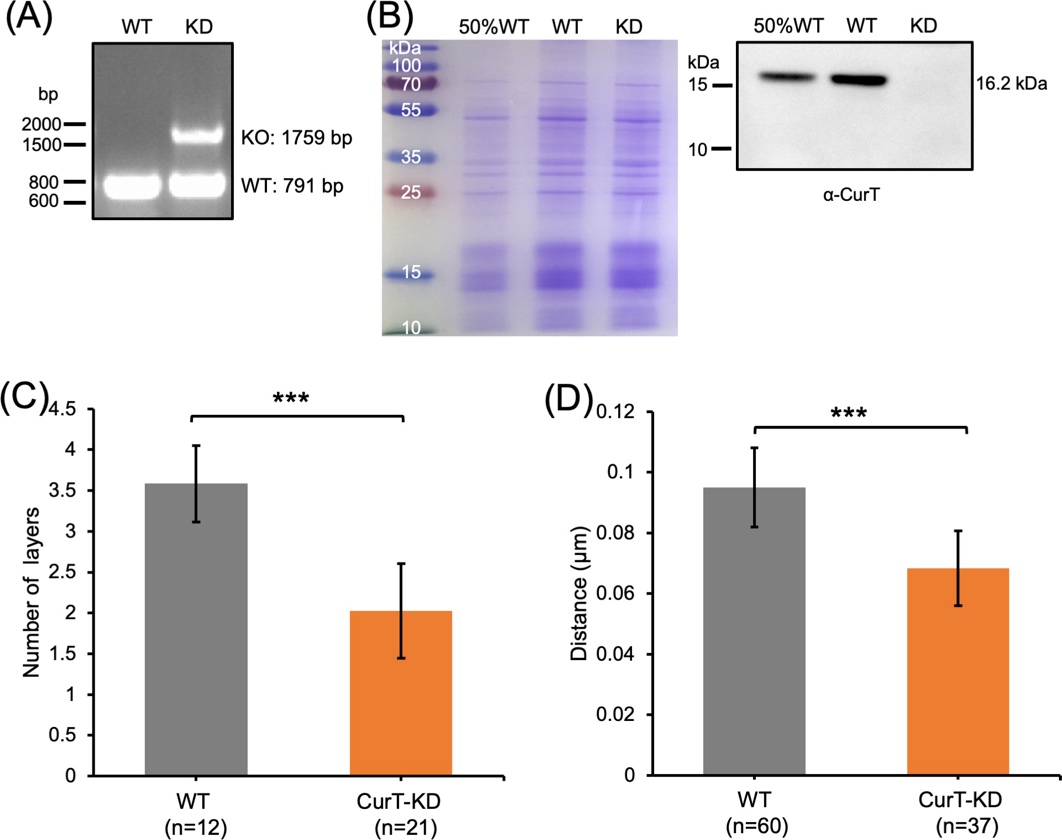


**Figure** **S3. Characteristics of the CurT knockdown (CurT-KD) strains grown under moderate light.** (**A)** PCR verification of the segregation of CurT-KD strains. (**B)** SDS-PAGE and immunoblot analysis of extracted thylakoids revealed that CurT knockdown resulted in a significantly low level of CurT in Syn7942, which was undetectable using α-CurT. **(C)** Counts of thylakoid membrane layers per cell of WT and CurT-KD. An incomplete layer of the thylakoid membrane is counted as 0.5. (**D)** Measurements of the distance between two adjacent layers of thylakoid membranes of WT and CurT-KD. Data are shown as means ± SD. Significant differences were determined with Student’s t-test. *: 0.01 < *p* < 0.05, **: 0.001 < *p* < 0.01, ***: *p* < 0.001.


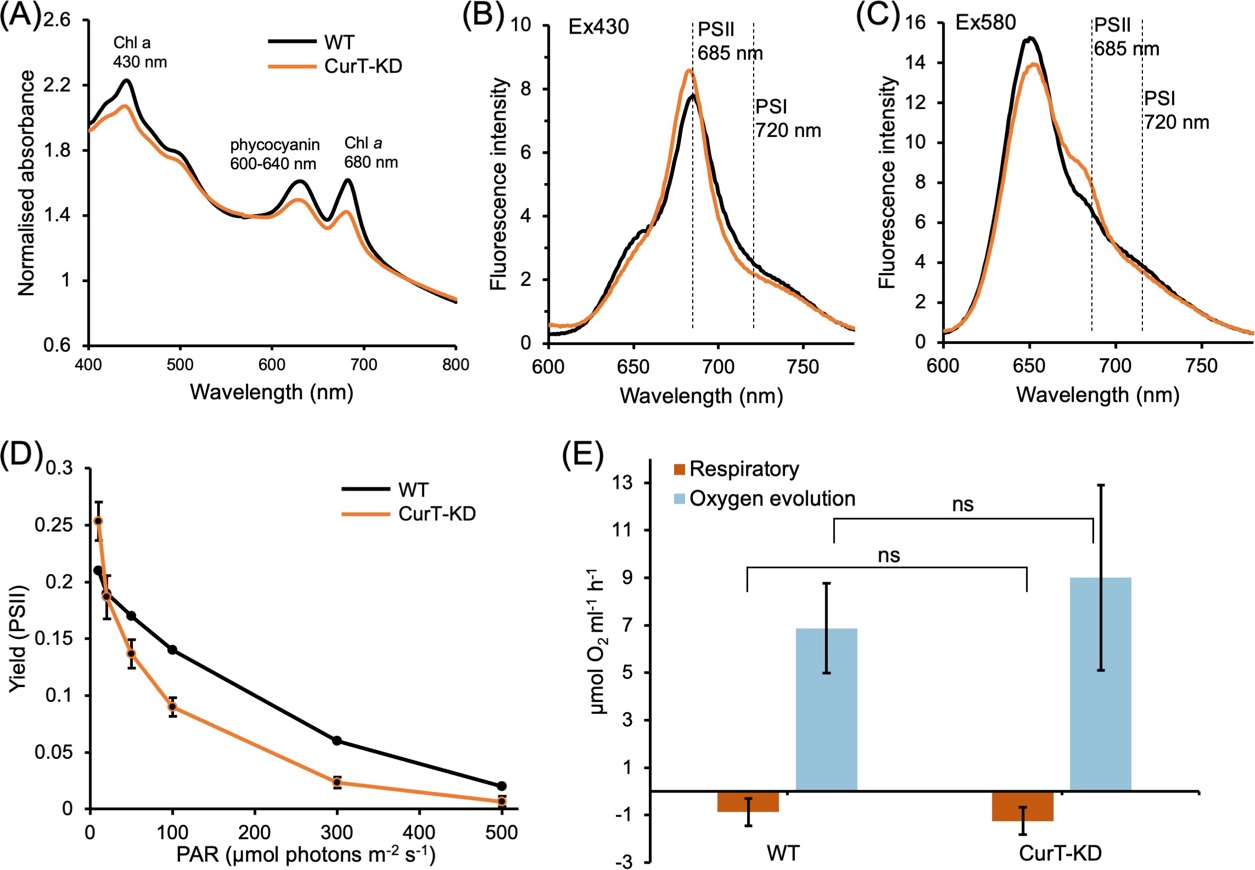


**Figure S4. Physiological characterization of the CurT-KD** **strain grown under moderate light. (A)** Absorption spectra of WT and CurT-KD from 400 nm to 800 nm normalized at OD_750_. (**B)** Room temperature fluorescence emission spectra of WT and CurT-KD, when excited at 430 nm (chlorophylls), normalized at OD_750_. (**C)** Room temperature fluorescence emission spectra of WT and CurT-KD, when excited at 580 nm (phycobilisomes), normalized at OD_750_. (**D)** The effective quantum yield of PSII (ΦPSII) as a function of photosynthetically active radiation (PAR) (chlorophyll *=* 20 μM). (**E)** Oxygen evolution and respiratory rates of WT and CurT-KD, with chlorophyll *a* level adjusted to 20 mM. The oxygen evolution was measured with a red LED light source peak at 650 nm. ns (not significant): *p* > 0.05.

**
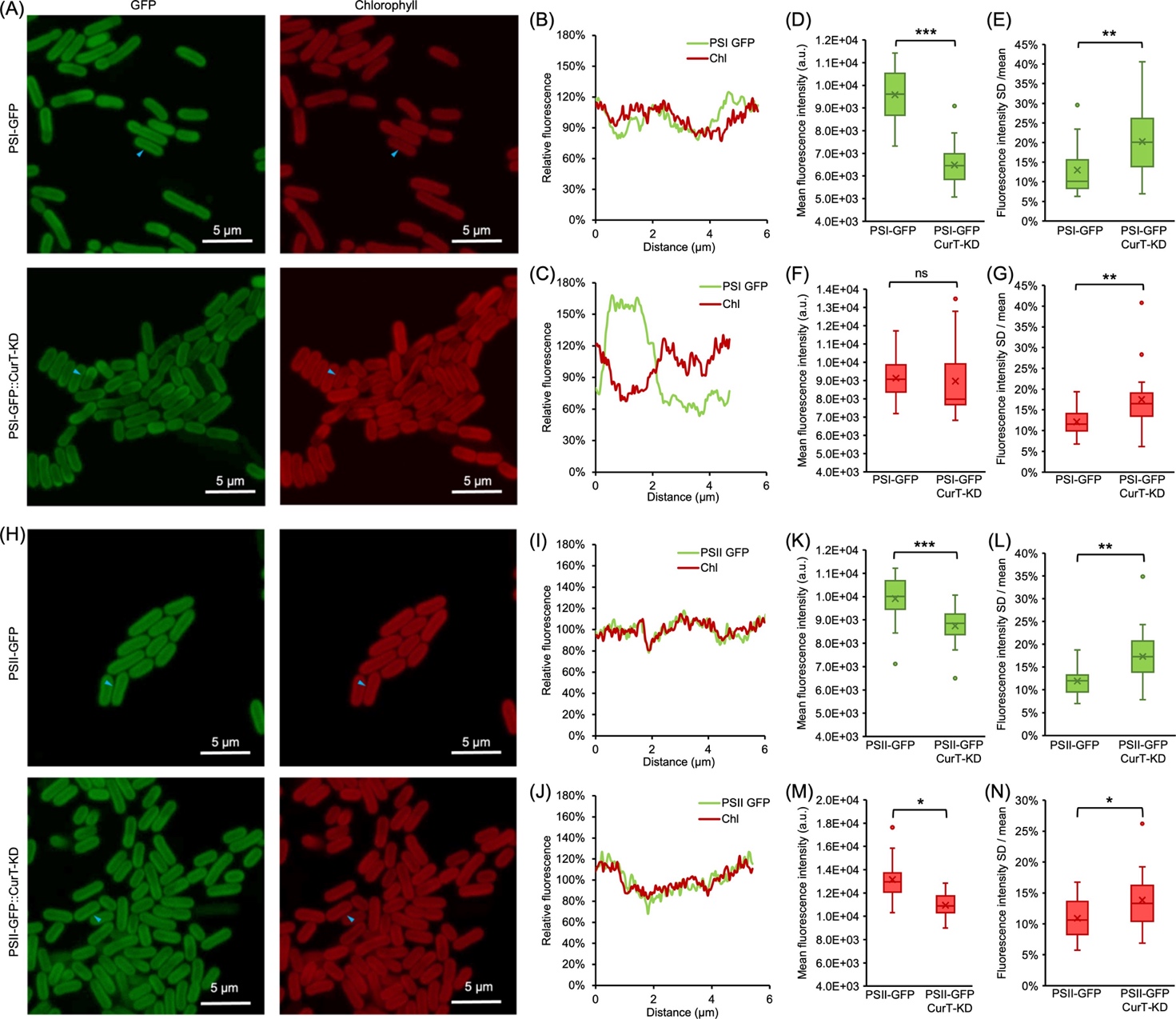
**

**Figure S5. Confocal microscopy imaging and characterisation of PSI-GFP cells and PSI-GFP::CurT-KD cells grown under moderate light. (A)** Confocal microscopy imaging of PSI-GFP and PSI-GFP::CurT-KD cells was performed in two channels—500–520 nm for GFP fluorescence (green) and 670–720 nm for chlorophyll *a* fluorescence (red). (**B, C)** Plot profiles of GFP and chlorophyll fluorescence intensities in the thylakoid membranes of PSI-GFP and PSI-GFP::CurT-KD cells (panel A, blue arrows), normalized to the mean fluorescence intensity of each cell. (**D, F)** present the mean GFP and chlorophyll fluorescence intensities along the thylakoid membranes, with values in arbitrary units (a.u.) (*n* = 20 for each group). (**E, G)** Analysis of the standard deviation (SD) of GFP and chlorophyll fluorescence intensities between the PSI-GFP and PSI-GFP::CurT-KD strains, with SD values normalized to the mean (*n* = 20 for each group). (**H-N)** Analysis of the fluorescence profiles, mean fluorescence intensity, and SD comparison of the PSII-GFP and PSII-GFP::CurT-KD cells. The plots shown in panels I and J correspond to the cells indicated by blue arrows in panel H. Data are shown as means ± SD, with significant differences determined using Student’s t-test (ns: *p* > 0.05, *: 0.01 < *p* < 0.05, **: 0.001 < *p* < 0.01, ***: *p* < 0.001).

**Table S1. Antibodies used in this study.**

| Antibody name | Manufacturer | Cat. No. | Dilution | Loading amount (μg total protein) | Protein size  (kDa) |
| --- | --- | --- | --- | --- | --- |
| Anti PsaB (rabbit) | Agrisera | AS10 695 | 1:1000 | 15 | 81.48 |
| Anti PsbA1 (rabbit) | Agrisera | AS05 084 | 1:10000 | 15 | 39.97 |
| Anti PsbD (rabbit) | Agrisera | AS06 146 | 1:5000 | 15 | 39.45 |
| Anti PetC (rabbit) | Agrisera | AS08 330 | 1:5000 | 40 | 18.83 |
| Anti AtpB (rabbit) | Agrisera | AS05 085 | 1:5000 | 25 | 51.7 |
| Anti IsiA (rabbit) | Agrisera | AS06 111 | 1:1000 | 60 | 36.98 |
| Anti RbcL (rabbit) | Agrisera | AS03 037 | 1:1000 | 30 | 52.45 |
| Anti CurT (rabbit) | (Heinz, Rast et  al. 2016). |  | 1:1000 | 80 | 16.21 |
| Goat anti rabbit | Agrisera | AS09 602 | 1:10000 | 2^nd^ antibody | 2^nd^ antibody |

**Supplementary references:**

1. Gust B, Kieser T, Chater K. REDIRECT technology: PCR-targeting system in *Streptomyces coelicolor*. The John Innes Centre, Norwich, United Kingdom. 2002.

2. Gust B, Chandra G, Jakimowicz D, Yuqing T, Bruton CJ, Chater KF. λ Red-mediated genetic manipulation of antibiotic-producing Streptomyces. *Adv Appl Microbiol*. 2004;54:107-28.

3. Casella S, Huang F, Mason D, Zhao G-Y, Johnson GN, Mullineaux CW, et al. Dissecting the native architecture and dynamics of cyanobacterial photosynthetic machinery. *Mol Plant*. 2017;10 (11):1434-48.

4. Heinz S, Rast A, Shao L, Gutu A, Gügel IL, Heyno E, et al. Thylakoid membrane architecture in synechocystis depends on CurT, a homolog of the granal CURVATURE THYLAKOID1 proteins. *Plant Cell*. 2016;28 (9):2238-60.

5. Ritchie RJ. Consistent sets of spectrophotometric chlorophyll equations for acetone, methanol and ethanol solvents. *Photosynth Res*. 2006;89 (1):27-41.

6. Cameron JC, Wilson SC, Bernstein SL, Kerfeld CA. Biogenesis of a bacterial organelle: the carboxysome assembly pathway. *Cell*. 2013;155 (5):1131-40.

7. Lobley A, Whitmore L, Wallace B. DICHROWEB: an interactive website for the analysis of protein secondary structure from circular dichroism spectra. *Bioinformatics*. 2002;18 (1):211-2.

8. Whitmore L, Wallace B. DICHROWEB, an online server for protein secondary structure analyses from circular dichroism spectroscopic data. *Nucleic Acids Res*. 2004;32 (suppl_2):W668-W73.

9. Whitmore L, Wallace BA. Protein secondary structure analyses from circular dichroism spectroscopy: methods and reference databases. *Biopolymers*. 2008;89 (5):392-400.

10. Provencher SW, Gloeckner J. Estimation of globular protein secondary structure from circular dichroism. *Biochemistry*. 1981;20 (1):33-7.

11. Van Stokkum IH, Spoelder HJ, Bloemendal M, Van Grondelle R, Groen FC. Estimation of protein secondary structure and error analysis from circular dichroism spectra. *Anal Biochem*. 1990;191 (1):110-8.

12. Huang F, Kong W, Sun Y, Chen T, Dykes GF, Jiang YL, et al. Rubisco accumulation factor 1 (Raf1) plays essential roles in mediating Rubisco assembly and carboxysome biogenesis. *Proc Natl Acad Sci USA*. 2020;117 (29):17418-28.

13. Sun Y, Wollman AJM, Huang F, Leake MC, Liu LN. Single-organelle quantification reveals the stoichiometric and structural variability of carboxysomes dependent on the environment. *Plant Cell*. 2019;31 (7):1648-64.

14. Huang F, Vasieva O, Sun Y, Faulkner M, Dykes GF, Zhao Z, et al. Roles of RbcX in carboxysome biosynthesis in the cyanobacterium *Synechococcus elongatus* PCC7942. *Plant Physiol*. 2019;179 (1):184-94.

15. Sun Y, Casella S, Fang Y, Huang F, Faulkner M, Barrett S, et al. Light modulates the biosynthesis and organization of cyanobacterial carbon fixation machinery through photosynthetic electron flow. *Plant Physiol*. 2016;171 (1):530-41.

16. Rumbaugh G, Miller CA. Epigenetic changes in the brain: measuring global histone modifications. *Methods Mol Biol*. 2011;670:263-74.

17. Yang J, Yan R, Roy A, Xu D, Poisson J, Zhang Y. The I-TASSER Suite: protein structure and function prediction. *Nat Methods*. 2015;12 (1):7-8.

18. Roy A, Kucukural A, Zhang Y. I-TASSER: a unified platform for automated protein structure and function prediction. *Nat Protoc*. 2010;5 (4):725-38.

19. Zhang Y. I-TASSER server for protein 3D structure prediction. BMC Bioinf. 2008;9 (1):40.

20. Abramson J, Adler J, Dunger J, Evans R, Green T, Pritzel A, et al. Accurate structure prediction of biomolecular interactions with AlphaFold 3. *Nature*. 2024;630 (8016):493-500.

21. Miles AJ, Wallace BA. Circular dichroism spectroscopy of membrane proteins. *Chem Soc Rev*. 2016;45 (18):4859-72.

22. Drin G, Antonny B. Amphipathic helices and membrane curvature. *FEBS Lett*. 2010;584 (9):1840-7.

23. McMahon HT, Boucrot E. Membrane curvature at a glance. *J Cell Sci*. 2015;128 (6):1065-70.

24. Edelmaier CJ, Klawa SJ, Mofidi SM, Wang Q, Bhonge S, Vogt EJD, et al. Charge distribution and helical content tune the binding of septin’s amphipathic helix domain to lipid membranes. Biophys J. 2025;124 (8):1298-1312.

25. Cui H, Lyman E, Voth GA. Mechanism of membrane curvature sensing by amphipathic helix containing proteins. *Biophys J*. 2011;100 (5):1271-9.

26. Rubin BE, Wetmore KM, Price MN, Diamond S, Shultzaberger RK, Lowe LC, et al. The essential gene set of a photosynthetic organism. *Proc Natl Acad Sci USA*. 2015;112 (48):E6634-E43.

27. Price MN, Wetmore KM, Waters RJ, Callaghan M, Ray J, Liu H, et al. Mutant phenotypes for thousands of bacterial genes of unknown function. *Nature*. 2018;557 (7706):503-9.

28. Armbruster U, Pribil M, Viola S, Xu W, Scharfenberg M, Hertle AP, et al. Arabidopsis CURVATURE THYLAKOID1 proteins modify thylakoid architecture by inducing membrane curvature. *Plant Cell*. 2013;25 (7):2661-78.

29. Huokko T, Ni T, Dykes GF, Simpson DM, Brownridge P, Conradi FD, et al. Probing the biogenesis pathway and dynamics of thylakoid membranes. *Nat Commun*. 2021;12 (1):1-14.

30. Liberton M, Page LE, O'Dell WB, O'Neill H, Mamontov E, Urban VS, et al. Organization and flexibility of cyanobacterial thylakoid membranes examined by neutron scattering. *J Biol Chem*. 2013;288 (5):3632-40.

31. Zhang Z, Zhao L-S, Liu L-N. Characterizing the supercomplex association of photosynthetic complexes in cyanobacteria. *R Soc Open Sci*. 2021;8 (7):202142.

32. Khrouchtchova A, Hansson M, Paakkarinen V, Vainonen JP, Zhang S, Jensen PE, et al. A previously found thylakoid membrane protein of 14 kDa (TMP14) is a novel subunit of plant photosystem I and is designated PSI‐P. *FEBS Lett*. 2005;579 (21):4808-12.

33. Wang Q, Jantaro S, Lu B, Majeed W, Bailey M, He Q. The high light-inducible polypeptides stabilize trimeric photosystem I complex under high light conditions in Synechocystis PCC 6803. *Plant Physiol*. 2008;147 (3):1239-50.

34. Ostermeier M, Heinz S, Hamm J, Zabret J, Rast A, Klingl A, et al. Thylakoid attachment to the plasma membrane in Synechocystis sp. PCC 6803 requires the AncM protein. *Plant Cell*. 2022;34 (1):655-78.
